# Uterine Artery Embolization for Treatment of Symptomatic Fibroids: A Review of the Evidence

**DOI:** 10.5812/ircmj.16699

**Published:** 2013-12-05

**Authors:** Kavous Firouznia, Hossein Ghanaati, Amir Hossein Jalali, Madjid Shakiba

**Affiliations:** 1 Advanced Diagnostic and Interventional Radiology Research Center, Tehran University of Medical Sciences, Tehran, IR Iran

**Keywords:** Uterine Artery Embolization, Angiography, Complications, Uterine Artery Embolization

## Abstract

Fibroids are the most common benign tumors of the uterus during female reproductive age. Uterine artery embolization (UAE) using embolic particles (PVA, Gelfoam) to occlude the uterine arteries, have been reported as a relatively safe, effective, and durable nonsurgical alternative to hysterectomy in diminishing fibroid-related symptoms. To block the arterial blood supply to the fibroid completely, UAE is typically performed in both uterine arteries by an experienced interventional radiologist. Reduction in menorrhagia has been reported as 80-93 percent and the mean decrease in fibroid size varies from 50-78% in the literature. In our center improvement in menstrual bleeding after 6 months was 80.3%, and uterine fibroids underwent shrinkage of 63.7±33.7% after12 months. Complication rate including amenorrhea ranges from 1% - 7% in the literature. UAE may be followed by menopause in 1% of cases. Nevertheless, it is usually encountered in women in their late 40s. It seems that the future of UAE depends on optimal selection of patients according to volume-shrinkage prediction and fertility outcome. Although pregnancy is possible after embolization, however neither fertility preservation nor improvement can be guaranteed following UAE. Indeed, Women who desire to become pregnant should be cautioned about potential complications during pregnancy. The aim of this review is to discuss about the efficacy, safety, technique, and choice of embolic agent. Also we present the effects of this technique on fertility and pregnancy outcome and also methods for dose reduction during this procedure.

## 1. Background

Uterine artery embolization (UAE) has been accepted as a safe and effective alternative treatment for symptomatic fibroids firstly reported by Ravina in 1995 (1). Uterine fibroids (also called leiomyoma or myoma) are one of the most common benign tumors which occur in about 40% of women by age 35 years (2). Fibroids constitute a major health care problem and about 30% of more than 600/00 hysterectomies performed annually in the USA are due to fibroids (3). Although many of women with fibroids are asymptomatic, some of them have annoying symptoms. These symptoms include prolonged and heavy menstrual bleeding, pelvic pain, pelvic pressure, urinary frequency and reproductive dysfunction. The costs for annual health care for a woman with uterine fibroids are 3.2 times higher than a woman without fibroid in united stated (4). The diagnosis of fibroids usually is suspected by the palpation of enlarged uterus and confirmed by pelvic ultrasound. In the diagnosis of uterine fibroids, ultrasonography is as efficient as MRI, although MRI shows a better resolution of fibroids and therefore better fibroid mapping. Indeed, MRI with and without contrast is useful for evaluation of the signal intensity changes before and after embolization (5). Conventional treatment options for uterine fibroids (hysterectomy, myomectomy and hormonal therapy) have substantial advantage and disadvantages. Laparoscopic uterine artery occlusion, Doppler – guided uterine artery occlusion, and MR-focused ultrasound surgery are other less invasive procedures for uterine fibroids. In this review we provide an overview of patient selection, indications, technique, complications, outcomes, pregnancy, and dose reduction in UAE for symptomatic fibroids.

## 2. Patient Selection

### 2.1. Preprocedure Evaluation

The preprocedural assessment should include a cooperative relationship between the gynecologist and interventional radiologist. This cooperation should be followed for post procedure care and long-term follow up (6). The decision to treat should be based patient’s symptoms including pelvic pain or pressure, urinary frequency, menstrual history and also imaging findings. In some centers MRI is preferred imaging method due to better resolution, better localization of fibroid localization and also post contrast enhancement in comparison to ultrasonography (7).

### 2.2. Indications

It seems that a large number of patients with symptomatic fibroids are potentially suitable for UAE. Also in women who have asymptomatic fibroids, UAE usually is not suitable method and other treatments should be considered. In most women who have uterine fibroids, heavy menstrual bleeding is indicated for UAE. Those who have not responded to other therapies are potential candidates for UAE. Repeat surgery due to adhesion formation is technically complicated for those previously performed myomectomy.

Preoperative MRI can be useful for assessment artery supply that may impact treatment planning. A myoma which has high signal intensity on T1-weighted images and shows no enhancement after Gd injection is unlikely to improve after UAE (8).

### 2.3. Contraindications

UAE is contraindicated in patients with renal failure unless the patient is undergoing dialysis, severe prior anaphylactic reactions, and also pregnancy. Patients who have pelvic infection are contraindicated due to risks of septic uterus (8). Other typical exclusion criteria are: history of pelvic radiation, acute vasculitis, pelvic malignancies, and the presence of immune-compromised condition (9). Indeed suspicious adenexal masses should be evaluated before UAE.

### 2.4. Diagnosis and Imaging Techniques

Subsequent to symptom evaluation, diagnosis of fibroids is usually made via Gynecologist by palpation of enlarge uterus. As history and physical examination have limitations and some conditions such as adenexal masses, adenomyosis, polyps, and cancers may mimic the fibroid related symptoms; fibroids must be confirmed with imaging. Imaging has an essential role in the evaluation, treatment and management of those women who underwent UAE. A number of imaging modalities such as ultrasonography and MRI are used for this purpose, and each method potentially has advantages and disadvantages. Ultrasound is the first technique for the evaluation of fibroids, and the preferred ultrasonography method is transabdominal. A linear probe with low to medium frequency (3.5-5 MHz) is suitable for this purpose.

Transvaging ultrasonography may be useful for better depiction of endometrium, and in such cases 5-7 MHz transvaginal probes provide better spatial resolution (10). Using color Doppler sonography we can reveal the vascularity of the fibroids and uterus and its follow patterns (11). There is marked peripheral blood flow with decreasing central flow in uterine fibroids (12). In one study Fleischer and his coworkers, the authors found that hypervascular fibroids in 3 D color Doppler sonography have more reduction in size than isovascular or hypovascular fibroids after UAE (13). MRI is an accurate and noninvasive diagnostic modality for diagnosis, characterization and enumeration of uterine fibroids and also differentiation from other pelvic disorders such as adenomyosis. Multiplanner capabilities and also enhancement, not only detects fibroids, but also may predict who will benefit from the embolization. MRI due to its multiplanner capabilities is very useful in the evaluation of uterus after UAE for fibroid infarction, size change, persistent enhancement, recurrence, and also complications (14).

### 2.5. Technique of UAE and Choice of Embolization Agents

UAE is typically performed from a right femoral artery puncture using 4 or 5-Fr Cobra catheter (7). Aortography performs prior to pelvic arteriography with a pigtail catheter which placed in the abdominal aorta at the level of renal arteries. Selective catheterization of uterine artery usually completed using a microcatheter and in some centers like us it performs using the same cobra catheter (15). The tip of a 4- or 5-Fr cobra catheter should be positioned beyond the junction of the horizontal and descending portions of each uterine artery. We perform embolization using 500-710µm Polyvinyl alcohol (PVA) particles (contour, Boston scientific, Boston, MA, USA) injective manually under fluoroscopic control. The PVA particles slowly inject through the catheter and the injection stops upper cessation of arterial flow to avoid retrograde reflux of particle and infiltration to other internal artery side branches.

In circumstances that anastomoses encounter between the uterine and ovarian arteries, we position the catheter tip distal to the anastomosis and we use gelfoam particles to occlude the anastomosis temporarily and there after UAE will perform.

We make our gelfoam particles from sponge sheets (gelitaspon, Gelita Mwdical BV, Amesterdam , Netherlands). We cut the sheet into small fragments with scissors.

Finally, post embolization angiography should perform for the evaluation of redistribution.

There is no consensus about the choice of embolic agent. Small particles are reported to cause more shrinkage in fibroids but associated with higher damage to myometrium and also cervical or ovarian ischemia (16-18).

The technical aim of UAE is to release the embolic material into uterine arteries without permanent damage to uterine or other organs (19). In one study which conducted in 96 patients by katsumori et al., the authors concluded that gelatin sponge particle can cause long term symptom control for fibroid in most patients (20).

In another study they found complete devascularization in more than 80 % of patients after contrast enhanced MRI (21).

## 3. Periprocedural and Early Post Procedural Management

The most common immediate complication of UAE is pain, as a result of fibroid ischemia and myometrium ischemia. For this reason the patient undergoing UAE should be prepare for pelvic pain and also the symptoms of post embolization syndrome.

The pain usually peaks during two to six hours after the procedure and may be accompanied by nausea. Uterine cramping is relatively mild with proper management for the patients. Pain may be severe and usually controlled by nonsteroid anti-inflammatory drugs (19). In patients who not respond to oral analgesics, admition and parenteral analgesic prescription is very helpful (19). In EMMY trial, in-hospital opiates were administered in 64% of patients, but in some studies outpatient UAE has been recommended (22, 23). Postembolization syndrome usually circumscribes symptoms of anorexia, low-grade fever, malaise and fatigue (8). This syndrome should be distinguished from infection which lasts for more than 5 days.

## 4. Dose Reduction During UAE

All fluoroscopic procedures are accompanied by radiation exposure, thus reduction of the exposure dose to the ovaries is very important in UAE due to the patient’s age and the presence of ovaries in this location. The ovaries are very sensitive organs to radiation due to the direct path of the beam during embolization. Estimated ovarian dose after UAE is 30–100 times more than the trunk CT (0.1–1.9 cGy) and 12–30 times less than the dose after radiotherapy for pelvis’s Hodgkin disease (263–3,500 cGy) (24). The level of exposure associates with the tube voltage (Kv), current time of product (mAs), skin distance and the imaging methods (25).

Not only the experience of the interventionalist but also factors like collimation, number of projections and DSA image acquisition mode can cause radiation dose reduction (26, 27). Flat panel devices can increase the image quality and also reduce the radiation dose (28-30). In one randomized clinical trial study we enrolled 30 women who were candidate for UAE, and evaluated the ovarian doses after UAE in digital subtraction angiography (DSA) and digital flat-panel system (31). The mean right ovarian dose was 139.9±92 in DSA and 23.6±16.2 mGy in flat panel (P<0.0001), and these were 101.7±77.6 and 24.6±16.9 mGy in left ovary, respectively (P=0.002). In the other hand we found that the mean ovarian dose in the DSA group was about five times more than the flat panel group. Finally we have concluded that flat panel system can significantly reduce the radiation dose after UAE in comparison with DSA.

## 5. The effects of UAE on Fertility and Pregnancy Outcome

The leiomyomas are the most prevalent neoplasms during the fertility aged women that mostly develop in uterus (32) but can originate from smooth muscle tissues other than uterus (33). They are an important issue in infertility and abortion (48). As the fibroids are one of the reasons for repeated abortion and infertility, the treatment could be a solution for these patients. Fertility and pregnancy outcome after UAE is a controversial issue to date (35). In this regard, important and common patients’ questions include: “is it possible for me to become pregnant after UAE? Can UAE be considered a treatment for my infertility?” Thus the possibility of pregnancy after the UAE is the most important consideration. Many studies have reported the case series of successful pregnancies after UAE (35-41).

Redecha et al. (35) reported a prospective case series consisting 98 patients of myomas, in which 21 patients wished to become pregnant. Among these patients 6 had successful spontaneous conception (23.1%) In addition, their course of the pregnancies was uneventful and the patients did not have any serious complication. The mean weight of the newborns was 3,339 gr (35).

Walker et al. (18) reported a series of 56 completed pregnancies among approximately 1200 UAE (36). They reported premature delivery rate of 18%, a miscarriage rate of 30.4%, terminations rate of 5.4%, stillbirth rate of 3.6% and tubal ectopic pregnancy rate of 1.8%. Also they encountered a rate of bleeding in first, second and third trimester equal to 24%, 15% and 12%. The rate of premature rupture of membrane and IUGR were 9.1% and 3% respectively. Postpartum hemorrhage showed in 18% of the patients (36). Carpenter and Walker reported a series of 26 completed pregnancies among 671 women underwent UAE (37). Totally 27% ended in miscarriage while two patients had termination and one was ectopic pregnancy. Of 16 deliveries after 24 weeks, bleeding in first and second trimesters occurred in 40% and 33%, respectively. Twenty five percents had preterm deliveries and 88% give birth by caesarean section. Proteinuric hypertension was seen in 13% and two others encountered preterm spontaneous rupture of the membranes. Primary postpartum hemorrhage was seen in 20%. The average birth weight of term newborns was 3.39 kg and none of them was admitted in neonatal intensive care. There was 6.7% rate of IUGR (37).

Mara et al. (38) conducted a randomized clinical trial comparing UAE and myomectomy regarding midterm clinical and first reproductive results. They assessed 58 UAEs and 63 myomectomies. Comparing the obstetrical profile between two groups, they found a statistically higher rate of pregnancy and delivery in myomectomy group (78% vs. 50% and 48% vs. 19% respectively) while the abortion rate in UAE group was higher (64% vs. 23%). Mean birth weight (both greater than 3000 gr), frequency of postpartum hemorrhage, perinatal hypoxia, pre-eclampsia and fetal intrauterine growth restriction were statistically similar between two groups (38).

Goldberg et al in 2002 reviewed all pregnancy reports to date in addition of their two additional cases (totally 50 patients) (39). They showed “22% (11 of 49) rate of spontaneous abortion, a 17% (ﬁve of 29) rate of malpresentation, a 7% (two of 29) rate of small for gestational age infants, a 28% (nine of 32) rate of premature delivery, a 58% (18 of 31) cesarean delivery rate, and a 13% (four of 31) rate of postpartum hemorrhage”(39).

Firouznia et al. (40) in their case series studied 102 patients underwent UAE in which 23 were seeking pregnancy. Among these patients, 14 became pregnant (61%, nine were nulliparous). Totally two miscarriage happened and 13 others successfully completed an uncomplicated pregnancy by elective cesarean section. All of the neonates had Apgar scores greater than 8 and were healthy. The average weight of the neonates was 3,274±514.4 g and there was only one neonate with small for gestational age (weight 2,100 gr) (40).

Goldberg compared 53 pregnancies after UAE and 139 after laparoscopic myomectomy. They reported a higher rate of preterm delivery and malpresentation after UAE but the risk of postpartum hemorrhage and spontaneous abortion were statistically similar (42). According to the series of pregnancy reported after UAE, it can be concluded however some obstetrical complications are higher after UAE (in comparison to normal pregnancies and myomectomy and in a especial consideration, during first two years after treatment in comparison to myomectomy) (46, 49, 52, 53), the pregnancy is certainly possible after embolization and the newborn outcome seems normal and satisfactory (35, 40).

Some important point in this relation could be considered. First, it has been proposed the pregnancy complications are more probable in older women (45, 47). This is important when we realize the point that the many of fibroma treated patients who wants to be pregnant have been infertile and are older in comparison to the normal population. Another important point in relation to the obstetrical outcomes is the time between UAE and pregnancy. It is possible that if the pregnancy time is close to the UAE, the pregnancy complication rate could be higher. Many authors recommend a safe time of 2 years after UAE to become pregnant. Another point in these patients is the patients with myomatous uterus. Although the myomectomy seems the standard treatment for the fibroma patients who wants to be regnant, the UAE seems better option for the patients with myomatous uterine or for patients with multiple fibroids as the myomectomy will not be effective for these patients.

## 6. Future Advances

Some future advances seem to be enthusiastic in the field of UAE. First, introduction of new imaging techniques for better delineation of fibroids will be useful as these techniques could further classify the patients in to more homogeneous subgroups that make the possibility of more precise prognosis determination or even better treatment plans. For example, Fallahi et al. (48) in their study introduced a new uterine segmentation and volume measurement method for uterine fibroids on the MRI images that in comparison with manual segmentation method showed “mean similarity of 80%, mean sensitivity of 75.32% and a mean specificity of 89.5%”. In addition “the Pearson correlation coefficient between the areas measured by the manual method and the automated method was 0.99”(47). These results showed a good performance of the method for fibroid determination that could be useful for automated volumetric measurements. The use of volumetric analysis is not confined to this field and has been used commonly in other fields of the imaging (46).

Another field of interest in the UAE could be application of the other embolization agents such as glues instead of PVA particles. The assessment of the efficacy of these agents in comparison to the PVA would be of interest.

In addition, the assessment of long term follow up of patients treated with UAE and especially assessment of long term developmental profile of fetuses after UAE seems interesting.

## 6. Conclusions

UAE for fibroids is an effective and safe therapeutic modality for symptomatic fibroids especially for those patients who like to preserve their uterus.

This procedure needs no general anesthesia, no surgical incision, and no blood loss or transfusion. Recovery and time to return to normal activities is shorter than hysterectomy and open myomectomy.

Management of fibroids needs multidisciplinary approach with gynecologist and interventional radiologist according to the symptoms, the desire to preserve fertility and uterus. Other options for the treatment of fibroids should be evaluated in terms of efficacy, safety, and effect on the future fertility.
